# Correction: Causality Analysis: Identifying the Leading Element in a Coupled Dynamical System

**DOI:** 10.1371/journal.pone.0135511

**Published:** 2015-08-07

**Authors:** The PLOS ONE Staff

## Notice of Republication

This article was republished on July 24, 2015, to correct errors in to correct errors in Figs 2 and 4–8 that were introduced during the production process. The publisher apologizes for the errors. Please download this article again to view the correct version. The originally published, uncorrected article and the republished, corrected article are provided here for reference.

## Supporting Information

S1 File Originally published, uncorrected article. (PDF)

S2 File Republished, corrected article. (PDF)
