# Supplementary material for: Correction: Causality Analysis: Identifying the Leading Element in a Coupled Dynamical System
Source: PLoS One. 2015 Aug 7;10(8):e0135511. doi: 10.1371/journal.pone.0135511 (PMC4529231; doi:10.1371/journal.pone.0135511)
Supplement: S2 File — (PDF) [file pone.0135511.s002.pdf]

PLOS | ONE

RESEARCH ARTICLE

Causality Analysis: Identifying the Leading Element in a Coupled Dynamical System

Amir E. BozorgMagham[1]*, Safa Motesharrei[2,3,4], Stephen G. Penny[1,5], Eugenia Kalnay[1,4]

1 Department of Atmospheric and Oceanic Science (AOSC), University of Maryland, College Park, MD, 20742, USA, **2** National Socio–Environmental Synthesis Center (SESYNC), Annapolis, Maryland 21401, USA, **3** Department of Physics, University of Maryland, College Park, Maryland, 20742, USA, **4** Institute for Physical Science and Technology, University of Maryland, College Park, Maryland 20742, USA, **5** National Centers for Environmental Prediction (NCEP), College Park, MD 20740, USA

* amirb@atmos.umd.edu

CrossMark
click for updates

OPEN ACCESS

Citation: BozorgMagham AE, Motesharrei S, Penny SG, Kalnay E (2015) Causality Analysis: Identifying the Leading Element in a Coupled Dynamical System. PLoS ONE 10(6): e0131226. doi:10.1371/journal.pone.0131226

Editor: Daniele Marinazzo, Universiteit Gent, BELGIUM

Received: December 15, 2014

Accepted: May 30, 2015

Published: June 30, 2015

Copyright: © 2015 BozorgMagham et al. This is an open access article distributed under the terms of the Creative Commons Attribution License, which permits unrestricted use, distribution, and reproduction in any medium, provided the original author and source are credited.

Data Availability Statement: All relevant data are within the paper.

Funding: This work was supported by NASA 5-26630. The funders had no role in study design, data collection and analysis, decision to publish, or preparation of the manuscript.

Competing Interests: The authors have declared that no competing interests exist.

Abstract

Physical systems with time-varying internal couplings are abundant in nature. While the full governing equations of these systems are typically unknown due to insufficient understanding of their internal mechanisms, there is often interest in determining the leading element. Here, the leading element is defined as the sub-system with the largest coupling coefficient averaged over a selected time span. Previously, the Convergent Cross Mapping (CCM) method has been employed to determine causality and dominant component in weakly coupled systems with constant coupling coefficients. In this study, CCM is applied to a pair of coupled Lorenz systems with time-varying coupling coefficients, exhibiting switching between dominant sub-systems in different periods. Four sets of numerical experiments are carried out. The first three cases consist of different coupling coefficient schemes: I) Periodic–constant, II) Normal, and III) Mixed Normal/Non-normal. In case IV, numerical experiment of cases II and III are repeated with imposed temporal uncertainties as well as additive normal noise. Our results show that, through detecting directional interactions, CCM identifies the leading sub-system in all cases except when the average coupling coefficients are approximately equal, i.e., when the dominant sub-system is not well defined.

Introduction

Identifying the leading [sub–]system from a pair of coupled dynamical systems using only time–series is challenging when nothing or little is known about the underlying dynamics. The definitive approach to detect causal relationships between components of a system is to fully identify the underlying physical mechanisms and governing equations. However, we only have partial knowledge about the internal physical mechanisms in most cases and must resort to observed data to establish the existing causal relationships in such cases.

In linear systems, lead time of the driver (or equivalently, latency of the response) may indicate a causal relationship, which hence could be identified by [linear] lag–correlation analysis. In a nonlinear system, however, there may be no persistent lead–lag between the two signals,

for example, due to feedback loops between the variables. Therefore, a linear lag–correlation analysis may not reliably determine the correct causal relationship in a nonlinear system [1]. For example, Fig 1 shows lead–lag switching between two coupled variables. If one considers short intervals of the time–series and takes lead time and strong correlation as the indicators of causation, an incorrect conclusion about the causal relationship between the variables could be made. In such circumstances, one may analyze the signal phases to establish temporal precedence and exploit the phase slope index to estimate the flow direction of information flux [2].

The concept of information transfer has been used as an indication of causality. A practical measure of information transfer is the transfer entropy which distinguishes directional communications between variables of a system [3]. This probabilistic measure has been used in a variety of fields such as neuroscience [4, 5], the study of chemical processes [6], and cellular automata [7]. A more general form of the transfer entropy that has an augmented time–delay parameter can detect the propagation time in addition to the asymmetric information transfer between observed signals [8].

Before establishing the general notion of information transfer (and transfer entropy), a practical definition of causality was proposed by Wiener [9], and later adopted and formalized in terms of linear autoregression by Granger [10, 11]. In the context of information transfer, Granger causality is shown to be equivalent to transfer entropy for Gaussian variables [12]. Although the standard [linear] Wiener–Granger method was first introduced for economic systems, the method and its extended nonlinear versions gained widespread use in other fields such as neuroscience and finance [13–18]. According to the Wiener–Granger definition of causality, a candidate driver is among the drivers of a response signal if the response prediction error increases significantly by removing the candidate driver data from the universe of all drivers. More precisely, given sets of interdependent variables X and Y, it is said that "X causes Y" if, in an appropriate statistical sense, X assists in predicting the future of Y beyond the degree to which Y already predicts its own future [12]. The Granger method is a forward method as it uses the driver data to predict the response. An important characteristic of the Granger method is that it requires the signals to be separable and have non–zero entropy rates [19]. In a separable system, it is possible to separate a candidate driver's data from other factors, thus enabling prediction to be conducted using data sets including and excluding the candidate driver. Separability is a restrictive conditions since many observed signals of interest are from deterministic nonlinear systems with feedbacks between state variables, resulting in mixed information from different sources.

Although information transfer and Granger causality provide valuable statistical information about the observed signals and their asymmetric connectivity, "efficient" causal relationship, as defined by Lizier and Prokopenko, between variables of a system can ultimately be identified by interventional methods, i.e., perturbing the candidate cause variable to investigate its direct influence on the response variable [20, 21]. Information flow was proposed as a quantitative measure of intervention. This Bayesian probabilistic measure quantifies the distribution of a response variable as a result of "imposing" conditions. However, there are some practical limitations for applying this measure and detecting causal relationships in realistic systems. For example, one may need to know about the structure of the causal links in a network or the underlying rules of the causal interactions. But understanding such structures, not known *a priori*, is the purpose of a causality study. The back–door adjustment criterion was proposed to solve this problem under certain conditions [20–22].

Sugihara et al.[23] investigated the problem of causality from a new perspective, proposing the Convergent Cross Mapping (CCM) method for deterministic nonlinear systems with smooth manifolds. The fundamental idea of the CCM method is that if Y is causally influenced by X, then Y has signatures of X such that the historical record of Y can reliably estimate the

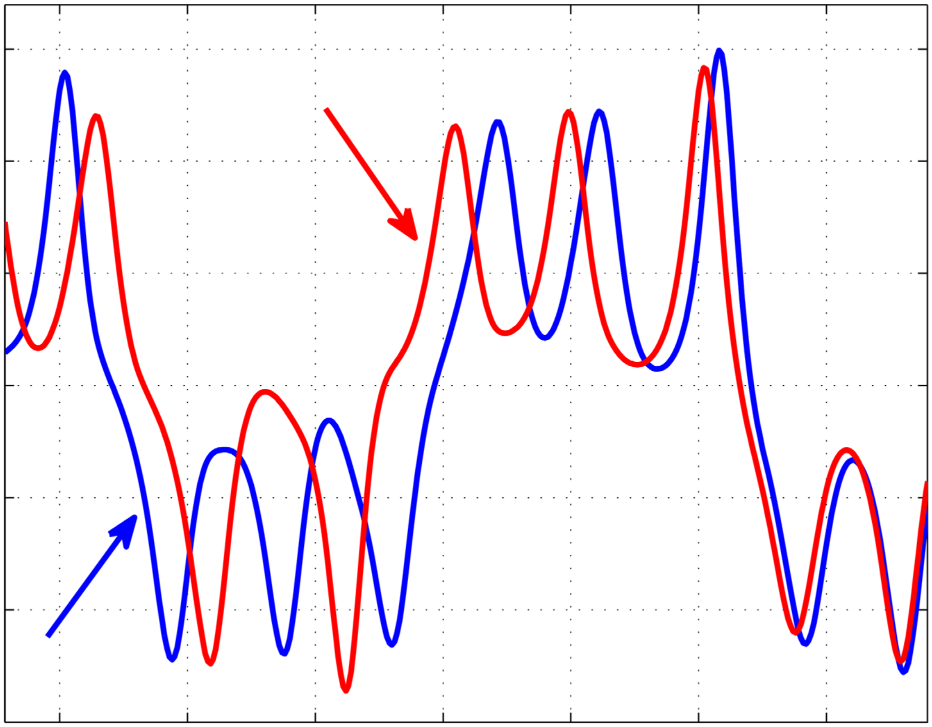

Fig 1. An example of lead–lag switching between two signals of a nonlinear system. Observation of the leading signals (shown by the colored arrows) in short intervals may result in an incorrect conclusion about the cause–effect relationship. The time–series in this figure are generated by Eq (9).

doi:10.1371/journal.pone.0131226.g001

state of X. Therefore, a better estimate of the driver variable, X, shows stronger causal influence on the response variable, Y.

It is important to note that: (i) unlike Granger causality that uses prediction as its fundamental basis, the CCM method is not based on the prediction of a variable; (ii) the CCM method does not apply Bayesian probability, which is the core concept of the transfer entropy and information flow measure; and (iii) the CCM method is not an interventional method. Therefore, it does not perturb the system to identify the micro–level causal effects in a system. It measures the correlation between the reconstructed and recovered manifolds of the observed signals (see §1).

Sugihara et al. [23] showed that CCM can identify unidirectional and bidirectional causation, and dominant driver, in weakly coupled nonlinear systems with constant coupling coefficients. They also considered [in Supplementary Materials] examples of systems with asymmetrical couplings, external forcing, and time delays [lagged influences]. They presented successful applications of the CCM method in ecology, biology, and geoscience [23–25]. Our study is motivated by the need to identify the dominant constituent in systems that are speculated to have time–varying interconnections with switching between the dominant elements in different periods. A challenging application is to identify the dominant variables of the global climate system from geophysical records of greenhouse gases concentrations and temperature proxy [26–28]. For this purpose, we consider coupled systems that (i) have variable coupling parameters in sequential periods, and (ii) the larger coupling coefficient is not fixed for a

specific sub–system. Thus, we may observe switching between the dominant sub–systems during successive periods. We investigate whether CCM, through detecting directional interactions, can identify the leading sub–system from a pair of coupled sub–systems under different conditions. In this paper, the leading sub–system is defined as the system with a larger average coupling coefficient over specified intervals of the time–series assuming that the coupled systems have the same time scales. To do that, we perform numerical experiments with different [linear] coupling schemes of Lorenz system [29] with asymptotic synchronization [30]. We consider periodic–constant (case I), normally distributed (case II), and mixed normally/non–normally distributed (case III) coupling coefficients. We also investigate the CCM results in the presence of temporal uncertainties and additive noise (case IV). Our results indicate that CCM can identify the leading sub–system, except when the average coupling coefficients are approximately equal, i.e., when the leading sub–system is not well-defined.

This article is organized as follows. Section §1 covers mathematical details of the CCM method. Section §2 describes the coupling schemes. In section §3, we show results of the numerical experiments. In section §4, we summarize and discuss our findings.

1 Method

In the CCM method, it is assumed that a response signal has signatures from its driver so that the approximate behavior of the driver can be estimated (or recovered) from the response signal. Thus, a better estimate of the driver shows stronger causal influence on the response variable. To implement the CCM method, we use the concept of one–to–one mapping between the original smooth manifold of the [full] system and the compact reconstructed phase spaces (shadowing manifolds) of the observed signals [31, 32]. If the two observed signals belong to the same dynamical system, a one–to–one mapping between the two reconstructed phase spaces could be established by considering the original manifold as an interface. If the dimension of the reconstructed phase spaces are selected based on the criteria of the false neighborhood method [33], arbitrary nearby points on the driver's reconstructed phase space map to the nearby points on the original attractor. Because of the causal influence of the driver, the nearby points on the original manifold stay close on the reconstructed phase space of the response signal. The stronger causal influence of the driver on the response, the closer the mapped points of the response signal on its shadowing manifold [23].

We assume, without loss of generality, that $x(t)$ is the driver and $y(t)$ is the response. The time–series $x(t)$ and $y(t)$ are sampled at equally spaced time intervals Δt,

$$\begin{cases} x_n = x(t) = x(t_0 + (n-1)\Delta t) \\ y_n = y(t) = y(t_0 + (n-1)\Delta t) \end{cases} \quad (1)$$

where $n = 1, 2, \cdots, N$, and N is the total number of data points in each time–series. We set $t_0 = 0$ to simplify the notation. We use $[x(t), y(t)]$ and $[x_n, y_n]$ notations interchangeably.

CCM uses the time–delay coordinates to reconstruct the phase spaces of the L-point windows from the time–series $x(t)$ and $y(t)$ [34]. The L–point windows are defined as

$$\begin{cases} W_x^{i,L} = \{x_i, x_{i+1}, \cdots, x_{i+L-1}\} \\ W_y^{i,L} = \{y_i, y_{i+1}, \cdots, y_{i+L-1}\} \end{cases} \quad (2)$$

for $i = 1$ to $i = N + 1 - L$ and $L_{min} \leqslant L \leqslant L_{max}$. We define L_{min} and L_{max} below. The L-point windows sweep the entire time–series $x(t)$ and $y(t)$ (see Fig 2a).

We use the average mutual information measure, I, a nonlinear generalization of the correlation function, to select the time lags τ as an integer multiple of Δt. This measure shows the

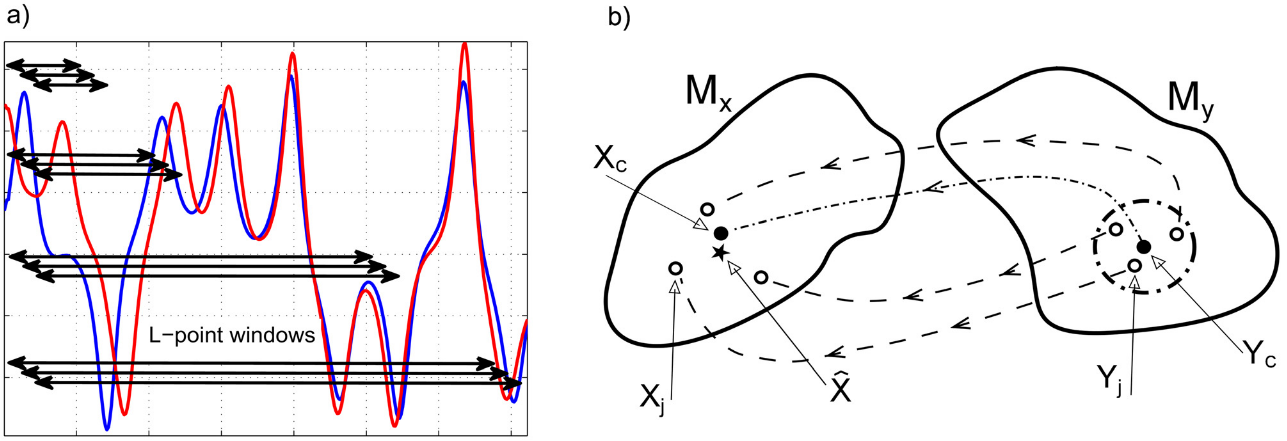

Fig 2. Schematics of *L*–point windows and reconstructed phase spaces. (a) Two time–series $x(t)$ and $y(t)$ and a schematic of the L–point windows with different lengths sweeping the whole span of the time–series. (b) A schematic of the reconstructed phase spaces of the L–point windows corresponding to two time–series. For each E-dimensional Y–central point, Y_c, in the response reconstructed phase space, M_y, a sufficient number of nearest neighbor points are selected (empty circles, Y_j, right) and their distances, d_j, to Y_c are determined. For each neighbor point, its contemporaneous point in the driver reconstructed phase space, M_x, is determined (empty circles, X_j, left). The weighted average of these points, $\hat{X}$, is compared with X_c, the true contemporaneous point in M_x corresponding to Y_c. The CCM coefficient, $\rho(L)$, is defined as the correlation coefficient between $\hat{X}$ and X_c, averaged over all possible L–point windows.

doi:10.1371/journal.pone.0131226.g002

average amount of information about a signal at $t + t'$, i.e., $s(t + t')$, when $s(t)$ is observed

$$I(t') = \sum_{n=1}^{N} P(s(t), s(t+t')) \log_2 \left[\frac{P(s(t), s(t+t'))}{P(s(t))P(s(t+t'))} \right], \tag{3}$$

where $P(s(t), s(t + t'))$ is the joint probability of measuring $s(t)$ and $s(t + t')$. We choose τ where the first minimum of $I(t')$ occurs [31, 35, 36].

We employ the false neighborhood method to determine the embedding dimension, E. The correct embedding dimension prevents self overlapping of the projected manifold. Therefore, "if E is qualified as an embedding dimension by the embedding theorem [31, 32], then any two points which stay close in the E-dimensional reconstructed space will be still close in the $(E + 1)$-dimensional reconstructed space. Such a pair of points are called true neighbors, otherwise, they are called false neighbors" [33].

By these two parameters, i.e., τ and E, we generate the time–delay coordinates as well as the reconstructed phase space resembling the original manifold of the attractor. The E-dimensional time–delay coordinate vectors corresponding to $W_x^{i,L}$ and $W_y^{i,L}$ are shown as

$$\begin{cases} m_x^{i,L}(t) = [x(t-(E-1)\tau), \cdots, x(t-\tau), x(t)] \\ m_y^{i,L}(t) = [y(t-(E-1)\tau), \cdots, y(t-\tau), y(t)] \end{cases} \tag{4}$$

for $t = (i - 1)\Delta t + (E - 1)\tau$ to $t = (i + L - 2)\Delta t$. Limits of t are chosen such that the first/last component of $m^{i,\,L}$ vectors corresponds to the first/last point of $W^{i,\,L}$ windows. The reconstructed phase spaces are

$$\begin{cases} M_x^{i,L} = \{m_x^{i,L}(t)\} \\ M_y^{i,L} = \{m_y^{i,L}(t)\} \end{cases} \tag{5}$$

each containing $L - (E - 1)(\tau/\Delta t)$ vectors.

After reconstructing the phase spaces, sufficient numbers of nearest neighbor points are selected for each E-dimensional vector in $M_y^{i,L}$, referred to as a Y–central point, Y_c (see Fig 2b). The neighbor points are denoted by Y_j, ordered by their distance, d_j, to Y_c, from nearest to farthest. This means Y_1 is the nearest neighbor to Y_c. The number of selected neighbor points should be equal or larger than $E + 1$ so the simplex method can represent the dynamics on an E-dimensional manifold [37]. This requirement specifies $L_{min} = (E + 2) + (E - 1)(\tau/\Delta t)$. We use the corresponding contemporaneous points of the Y_j in M_x, denoted by X_j, to calculate the position of a single point that represents them, $\hat{X}$. We define the exponentially decaying weights based on the distances between the Y_c and its neighbor points, d_j, as

$$\begin{cases} u_j = \exp\left(-\dfrac{\|d_j\|}{\|Y_c - Y_1\|}\right) \\ \omega_j = \dfrac{u_j}{\sum u_j} \end{cases} \tag{6}$$

where $\|\cdot\|$ is the Euclidean norm. The position of the representative point in M_x is calculated as

$$\hat{X} = \sum \omega_j X_j. \tag{7}$$

The set of these recovered points, $\{\hat{X}\}$, is the recovered phase space. If $x(t)$ and $y(t)$ are dynamically coupled, $\{\hat{X}\}$ should be strongly correlated to $M_x = \{X_c\}$, where X_c is the point corresponding to Y_c (see the star and black filled circle in Fig 2b). In addition, as L increases, the density of the points in the reconstructed phase space increases, hence the distances between the nearest neighbors shrink. Thus, $\hat{X}$ converges to a vicinity of X_c that becomes smaller as the causal effect of the driver increases. Therefore, the CCM coefficient, $\rho(L)$, as a measure of causal relationship between the [candidate] driver and the response signal is defined as the correlation coefficient between $\hat{X}$ and X_c, averaged over all possible L–point windows,

$$\rho_{X_c,\hat{X}|M_y}(L) = \left\langle \frac{\mathrm{cov}(\hat{X}, X_c)}{\sigma_{\hat{X}}\sigma_{X_c}} \right\rangle_L. \tag{8}$$

If there is a causal relationship between the signals, ρ converges to a constant equal or less than one. Note that $\langle \cdot \rangle_L$ indicates averaging over all L–point windows, and $\hat{X} \mid M_y$ emphasizes that $\hat{X}$ is estimated given M_y. If we study the opposite roles, i.e., $y(t)$ as the driver and $x(t)$ as the response, the CCM coefficient would be shown by $\rho_{Y_c,\hat{Y}|M_x}$. We simplify this notation by setting $\rho_{\hat{X}|Y} = \rho_{X_c,\hat{X}|M_y}$ and $\rho_{\hat{Y}|X} = \rho_{Y_c,\hat{Y}|M_x}$.

It is important to note that: (i) the CCM method, through detecting directional dynamical interactions, can investigate causal relationship over any window of the time–series that contains sufficient information about the attractor of the dynamical system. In the case of nonstationary signals, the CCM results during the considered time interval are presumably valid if the original manifold and the reconstructed phase spaces do not have significant changes, i.e., if the near–stationary condition holds. We do not discuss the application of CCM to nonstationary signals in this paper. (ii) CCM only considers the past data and does not attempt to predict the signals. Therefore, CCM could be robustly applied to chaotic systems, where predictions may rapidly diverge from the true/observed states. (iii) The identified causal relationship by the CCM method is not exclusive, i.e., the identified driver might be one of the many drivers of the system. Therefore, in systems with multiple variables, CCM can be applied and repeated

for different combinations of the candidate driver and response signals. (iv) Our results (not shown here) show that the CCM method is not sensitive to increases in the embedding dimension if it is chosen by the false neighborhood method. By increasing the embedding dimension beyond what is prescribed by the false neighborhood method, computation cost increases because the search process for the nearest neighbors should be performed in a larger space dimension. However, the CCM results remain almost unchanged because the extra dimensions do not add new information. In addition, the CCM results are not sensitive to small changes of τ if the embedding parameter remains in the vicinity of the first minimum of the average mutual information measure.

2 Experiment design with coupled Lorenz systems

In order to study the applicability of the CCM method to [strongly] coupled systems with variable coupling coefficients, we apply it to a pair of identical Lorenz systems [29], $\mathfrak{L}_X$ and $\mathfrak{L}_Y$, coupled with different coupling schemes. We choose to study synchronized dynamical systems, in which the coupling is canonically between two identical [sub–]systems coupled simultaneously with a linear coupling term where the dominant element is clearly identified by its larger coupling coefficient. Study of synchronized dynamical systems has expanded beyond the canonical case to included "generalized synchronization" [38], in which the two systems differ. Other work in synchronized dynamical systems has studied "lagged synchronization" [39]. Since this application of the CCM method is a new, we choose to focus on the canonical representation of the problem and allow for similar extensions in future work to address these more complex variations of the general problem.

The general form of $\mathfrak{L}_X$ and $\mathfrak{L}_Y$ is given by Eq (9).

$$\begin{aligned} \mathfrak{L}_X & \begin{cases} \dot{x}_1 = \sigma(x_2 - x_1) + k_\mu \mu(y_1) \\ \dot{x}_2 = x_1(\rho - x_3) - x_2 \\ \dot{x}_3 = x_1 x_2 - \beta x_3 \end{cases} \\ \mathfrak{L}_Y & \begin{cases} \dot{y}_1 = \sigma(y_2 - y_1) + k_\eta \eta(x_1) \\ \dot{y}_2 = y_1(\rho - y_3) - y_2 \\ \dot{y}_3 = y_1 y_2 - \beta y_3 \end{cases} \end{aligned} \tag{9}$$

The parameters of the Lorenz–63 systems are $(\sigma, \rho, \beta) = (16, 45.92, 4)$ throughout this paper. μ and η are the coupling coefficients, and k_μ and k_η are the binary on–off switches. Variations of the coupling coefficients, activation of the on–off switches, and duration of successive periods are discussed in section §3.

We investigate whether CCM can distinguish the leading system by detecting the difference between the CCM coefficients $\rho_{\hat{X}|Y}$ and $\rho_{\hat{Y}|X}$. We probe one signal from each system, $x_1(t)$ and $y_1(t)$ in the following experiments. When the two systems are strongly correlated, for example, due to a strong feedback loop, $\rho_{\hat{X}|Y}$ and $\rho_{\hat{Y}|X}$ are close to each other for large L's. (This is expected, unless the two systems have different amplitudes and time scales, as in the case of the ocean–atmosphere models [40].) Thus, one must investigate small differences between $\rho_{\hat{X}|Y}$ and $\rho_{\hat{Y}|X}$.

A brief description of the four experiments is as follows. In case I, $\mathfrak{L}_X$ and $\mathfrak{L}_Y$ are coupled by a periodic on–off switching mechanism and constant coupling coefficients during all periods. We cover 100 different combinations of coupling coefficients. In case II, both coupling coefficients in all periods are normal random variables with specified means and standard deviations.

In case III, one of the coupling coefficients is a normal random variable and the other one is from a Weibull (skewed non–normal) distribution [41]. In case IV, we consider the original pairs of signals from cases II and III and impose relative temporal shifts (representing temporal uncertainties) as well as additive Gaussian noise. In all experiments, we observe a strong correlation between the probed signals $x_1(t)$ and $y_1(t)$ due to bidirectional couplings in system Eq (9).

As discussed in §1, $\rho(L)$ converges to a constant by increasing the length of the L–point windows, provided that the two signals are dynamically connected. We test convergence of $\rho(L)$ over the range $L = 25$ to 500. We observe negligible variations in the CCM coefficients for $L \geq 300$. We choose $L = 500$ to report all the CCM coefficients. This choice of L is equivalent to 0.5 time unit since we sample the signals 1000 times per non–dimensional time unit.

3 Experiment results

Below we present the results of four sets of numerical experiments with the system Eq (9).

3.1 Case I: Coupled Lorenz systems, periodic–constant coupling coefficients. In this case, coupling coefficients μ and η assume constant integers 1 to 10, resulting in 100 combinations, during 10 consecutive periods of the time–series, each spanning 10 non–dimensional time units. The switching mechanism is controlled by k_μ and k_η, such that in odd periods $k_\mu = 0$ and $k_\eta = 1$ and in even periods $k_\mu = 1$ and $k_\eta = 0$ as described in Eq (10).

$$\begin{cases} (\eta, \mu) = \text{int}[1:10] \times \text{int}[1:10] \\ k_\eta = 1 \,\text{odd periods}, 0 \,\text{even periods} \\ k_\mu = 0 \,\text{odd periods}, 1 \,\text{even periods} \end{cases} \tag{10}$$

Fig 3a shows $\rho_{\hat{Y}|X}$ at $L = 500$ for different values of μ and η and random initial conditions. We observe similar patterns in $\rho_{\hat{Y}|X}$ and $\rho_{\hat{X}|Y}$ (not shown here) such as vertical and horizontal bands. For example, we see a blue horizontal band near $\mu = 1$ in Fig 3a, showing that the influence of $\mathfrak{L}_Y$ on $\mathfrak{L}_x$ is small. Therefore, $\{\hat{Y}\}$ (the recovered phase space of $\mathfrak{L}_Y$ from $\mathfrak{L}_X$), is poorly correlated to M_y (the reconstructed phase space of $\mathfrak{L}_Y$), hence the small values for $\rho_{\hat{Y}|X}$ on the band. Another observed pattern is the high values of ρ in the regions with the strongest coupling between the two sub–systems (high μ and η), showing the large mutual influence between the two systems.

To address the main question of this study we investigate $\rho_{\hat{Y}|X} - \rho_{\hat{X}|Y}$. We expect that if $\mathfrak{L}_Y$ is the leading sub–system, then $\rho_{\hat{Y}|X}$ is larger than $\rho_{\hat{X}|Y}$ and similarly, $\rho_{\hat{X}|Y}$ is larger than $\rho_{\hat{Y}|X}$ if $\mathfrak{L}_X$ is the dominant sub–system. Referring to Fig 3b, we observe that $\rho_{\hat{Y}|X} > \rho_{\hat{X}|Y}$ when $\mu > \eta$ (upper diagonal of Fig 3b) and $\rho_{\hat{X}|Y} > \rho_{\hat{Y}|X}$ when $\eta > \mu$ (lower diagonal of Fig 3b).

This numerical experiment with 100 combinations of coupling coefficients shows that even when ρ values are close to each other ($\rho_{\hat{Y}|X} \approx \rho_{\hat{X}|Y}$), CCM can capture the leading system through small differences between the CCM coefficients, except for $\mu \approx \eta$ where neither system is leading.

3.2 Case II: Coupled Lorenz systems, normally distributed coupling coefficients

In section §3.1, we studied CCM's capability to identify the leading system in coupled systems with periodic constant coupling coefficients. A more general and realistic case is a system with randomly distributed coupling coefficients and with random lengths of time periods. In this section, we choose the coupling coefficients from normal distributions. Note that these

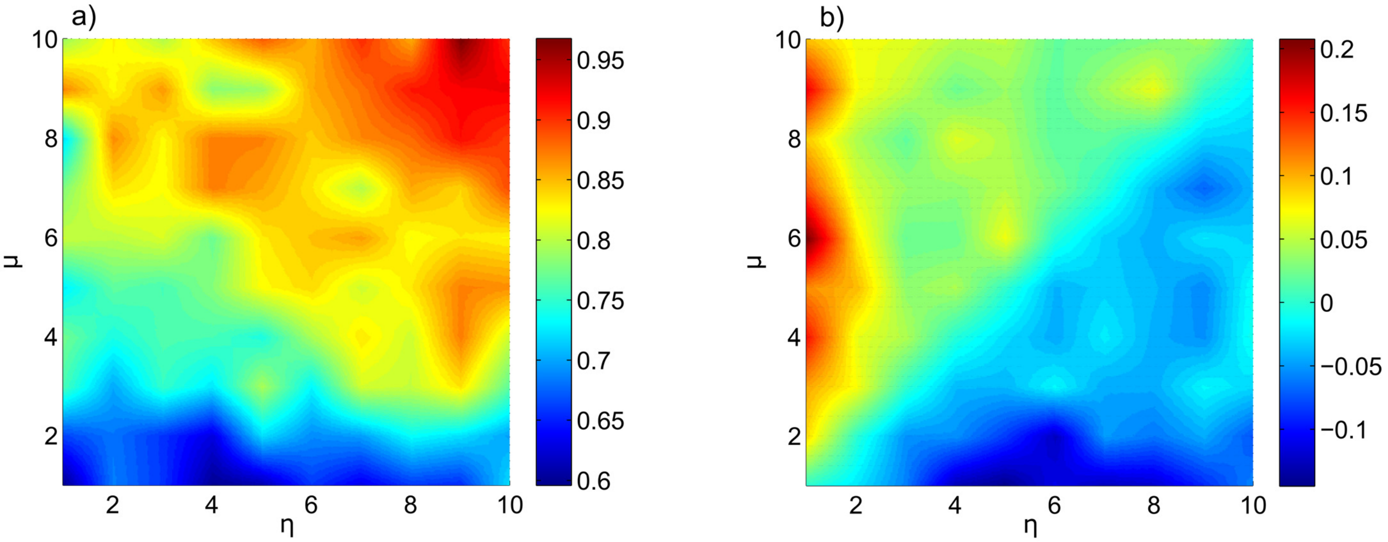

Fig 3. Case I: periodic–constant coupling coefficients. (a) $\rho_{\hat{Y}|X}$ at L = 500 over the range of μ and η given by Eq (10) and for random initial condition. (b) Difference between the CCM coefficients, $\rho_{\hat{Y}|X} - \rho_{\hat{X}|Y}$, at L = 500 over the specified range of μ and η.

doi:10.1371/journal.pone.0131226.g003

coefficients are constant during each period. In Eq (9), we set $\eta = \eta_N$ and $\mu = \mu_N$, where subscript N stands for normal random variables. We set the mean and standard deviation of the coupling coefficients such that the ratio of the averaged coupling coefficients $\bar{\eta}_N/\bar{\mu}_N$ covers the approximate range (0.5,5) and we have enough data points both above and below 1. The switching coefficients, k_η and k_μ, are kept on for all periods in order to have bidirectional communication between the two sub-systems. We also choose the duration of each period, T_i, from a normal distribution as described in Eq (11).

$$\begin{cases} k_\eta = 1, k_\mu = 1 \text{ for all periods} \\ \eta = \eta_N = \mathcal{N}(5.5, 2) \\ \mu = \mu_N = \mathcal{N}(\alpha, 2) \mid \alpha \in \text{int}[1:10] \\ T_i = \mathcal{N}(\bar{T} = 10, \sigma = 2) \mid i \in \text{int}[1:20] \end{cases} \tag{11}$$

Again, to address the main question of this study, we investigate the difference between the CCM coefficients, $\Delta\rho = \rho_{\hat{X}|Y} - \rho_{\hat{Y}|X}$, at L = 500 as a function of $\bar{\eta}_N/\bar{\mu}_N$, where $\bar{\eta}_N$ and $\bar{\mu}_N$ are the mean values of η_N and μ_N over the entire time–series. Fig 4a shows that the difference between the CCM coefficients is negative for $(\bar{\eta}_N/\bar{\mu}_N) < 1$ and positive for $(\bar{\eta}_N/\bar{\mu}_N) > 1$. We also observe a close–to–linear relationship between $\bar{\eta}_N/\bar{\mu}_N$ and $\Delta\rho$ in the considered range of the coupling coefficients.

We extend this analysis using the Monte Carlo method. We generate 1000 independent realizations of the coupled system for each $\alpha \in$ int [1:10] (thus, we have 10,000 individual data points) and calculate the difference between the CCM coefficients at L = 500. As we observe in Fig 4b, $\Delta\rho$ is negative for almost all realizations when $(\bar{\eta}_N/\bar{\mu}_N) < 1$ and positive when $(\bar{\eta}_N/\bar{\mu}_N) > 1$. Note that the distribution of the points on the two sides of $(\bar{\eta}_N/\bar{\mu}_N) = 1$ depends on the choices of η_N and μ_N in Eq (11). Fig 4a and 4b shows that CCM can determine the leading system when the coupling coefficients are normally distributed except when $\bar{\eta}_N \approx \bar{\mu}_N$, i.e., when the leading sub–system is not well–defined.

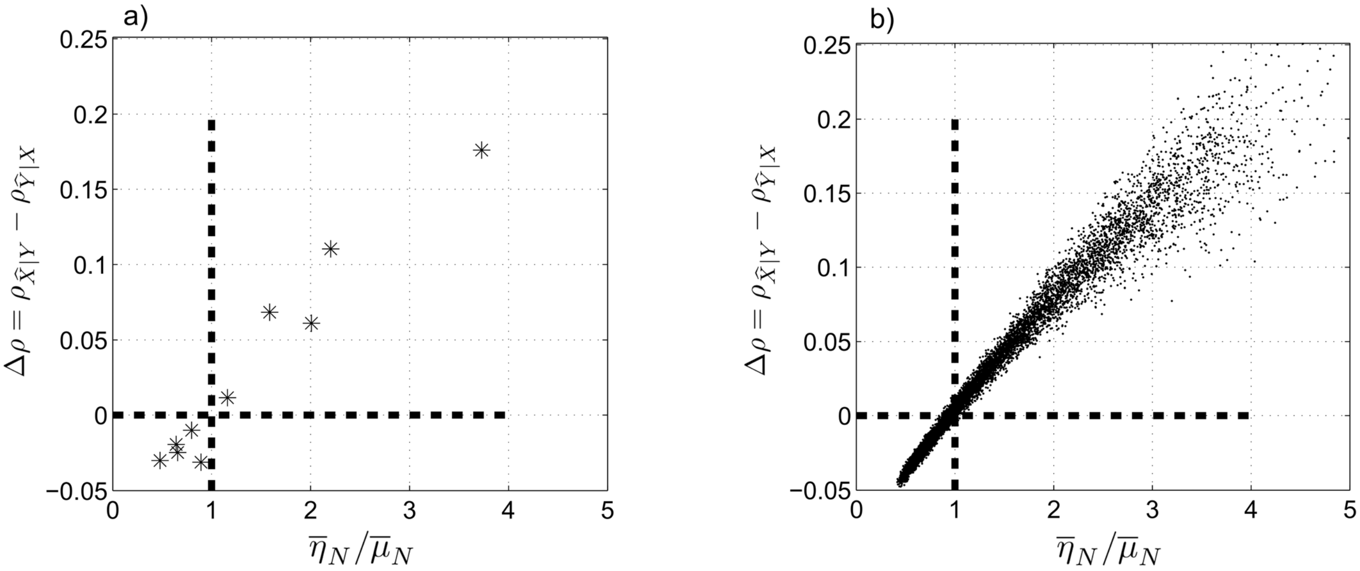

Fig 4. Case II: normally distributed coupling coefficients. (a) The difference between the CCM coefficients, $\Delta\rho = \rho_{\hat{X}|Y} - \rho_{\hat{Y}|X}$, at $L = 500$ as a function of the ratio of the averaged coupling coefficients, $\overline{\eta}_N/\overline{\mu}_N$. (b) Monte Carlo simulation of 1000 independent realizations of Eq (11) to calculate $\Delta\rho$ at $L = 500$ as a function of $\overline{\eta}_N/\overline{\mu}_N$. $\overline{\eta}_N$ and $\overline{\mu}_N$ are the mean values of η_N and μ_N over the span of the time–series. $\Delta\rho = 0$ and $\overline{\eta}_N/\overline{\mu}_N = 1$ are shown by dashed, bold lines.

doi:10.1371/journal.pone.0131226.g004

Because $\rho(L)$ is obtained by averaging over all possible L–point windows (see Eq (8)), one might be concerned whether the difference between ρ values, i.e., $\Delta\rho = \rho_{\hat{X}|Y} - \rho_{\hat{Y}|X}$, is statistically significant and meaningful. To address this concern, we perform a non–parametric one–sample Kolmogorov–Smirnov test for all data points of Fig 4a. Results of this test, for $\rho_{\hat{X}|Y}$ and $\rho_{\hat{Y}|X}$, fail to reject the null hypothesis that the data in the ρ vectors come from a standard normal distribution at 0.001 significance level. Next, a group t–test shows that for all the data points the obtained t–values exceed the required minimum corresponding to a critical p–value of 0.01. Therefore, we can conclude that the differences, $\Delta\rho$, are significant at 0.01 level. For example, for the smallest absolute value of $\Delta\rho$ in Fig 4a, the calculated t–value equals 3.16 which is larger than 2.58 corresponding to 0.01 critical p–value. These numerical values correspond to the sample size of the L–point windows at $L = 500$. Therefore, we can reject the null hypothesis that there is no significant difference between the mean values of ρ.

3.3 Case III: Coupled Lorenz systems, normally and non–normally distributed coupling coefficients

For numerical experiments §3.1 and §3.2, we considered constant and normally distributed coupling coefficients, respectively. In case II, the difference between the two coupling coefficients is also a normal variable because both coefficients are normal variables. In case III, to further generalize our study, we break the symmetry of the coupling coefficients by choosing one of them from a normal and the other from a non–normal random distribution. By this selection, the difference between the coupling coefficients is not a normal variable. We set $\eta = \eta_N$ as in §3.2 but choose $\mu = \mu_{Wb}$ from Weibull distributions [41]. As before, each time–series

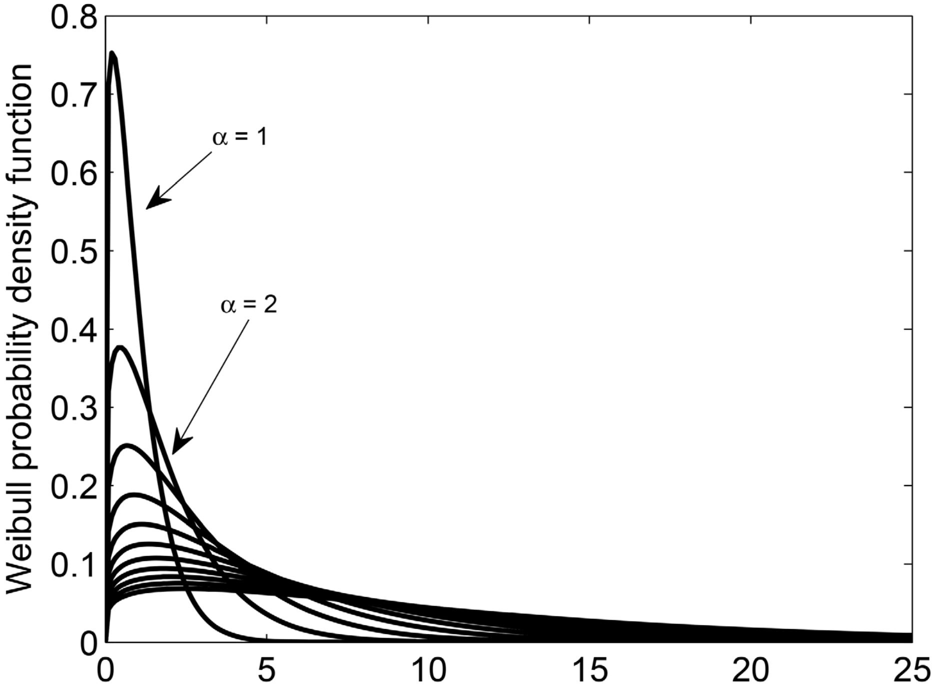

Fig 5. Weibull probability density function for $\alpha \in$ int [1:11] and shape factor equal to 1.2 in Eq 12.

doi:10.1371/journal.pone.0131226.g005

consists of twenty periods with normally distributed lengths. (During each period, the coupling coefficients remain constant.) The current setting is summarized in Eq (12).

$$\begin{cases} k_\eta = 1, k_\mu = 1 \text{ for all periods} \\ \eta = \eta_N = \mathcal{N}(5,2) \\ \mu = \mu_{Wb} = \text{Weibull}(\alpha, 1.2) \mid \alpha \in \text{int}[1:11] \\ T_i = \mathcal{N}(\bar{T} = 10, \sigma = 2) \mid i \in \text{int}[1:20] \end{cases} \tag{12}$$

Parameters of the Weibull distribution are chosen in order to have sufficient values of $\bar{\eta}_N/\bar{\mu}_{Wb}$ both below and above one. The same reasoning also applies to setting Eq (11) of experiment case II. Fig 5 shows the asymmetric Weibull probability density function for the parameters of Eq (12).

Similar to cases I and II, we investigate $\Delta\rho = \rho_{\hat{X}|Y} - \rho_{\hat{Y}|X}$ as a function of $\bar{\eta}_N/\bar{\mu}_{Wb}$ at $L = 500$ (see Fig 6a). Similar to Fig 4a, we observe that $\Delta\rho$ is negative for $(\bar{\eta}_N/\bar{\mu}_{Wb}) < 1$ and is positive for $(\bar{\eta}_N/\bar{\mu}_{Wb}) > 1$. This result supports our expectation about the ability of the CCM method to distinguish the leading sub–system.

We employ the Monte Carlo method to generate 1000 independent realizations of the coupled Lorenz systems with setting Eq (12) which results in a total of 11,000 individual data points. Then we calculate $\Delta\rho$ for each realization at $L = 500$, shown in Fig 6b. We again observe that $\Delta\rho$ is negative for $(\bar{\eta}_N/\bar{\mu}_{Wb}) < 1$ and positive for $(\bar{\eta}_N/\bar{\mu}_{Wb}) > 1$, except for a small fraction of points around $\bar{\eta}_N/\bar{\mu}_{Wb} \approx 1$ where the leading system is not well–defined. Therefore, CCM can distinguish the leading sub–system in a coupled system with normal/non–normal

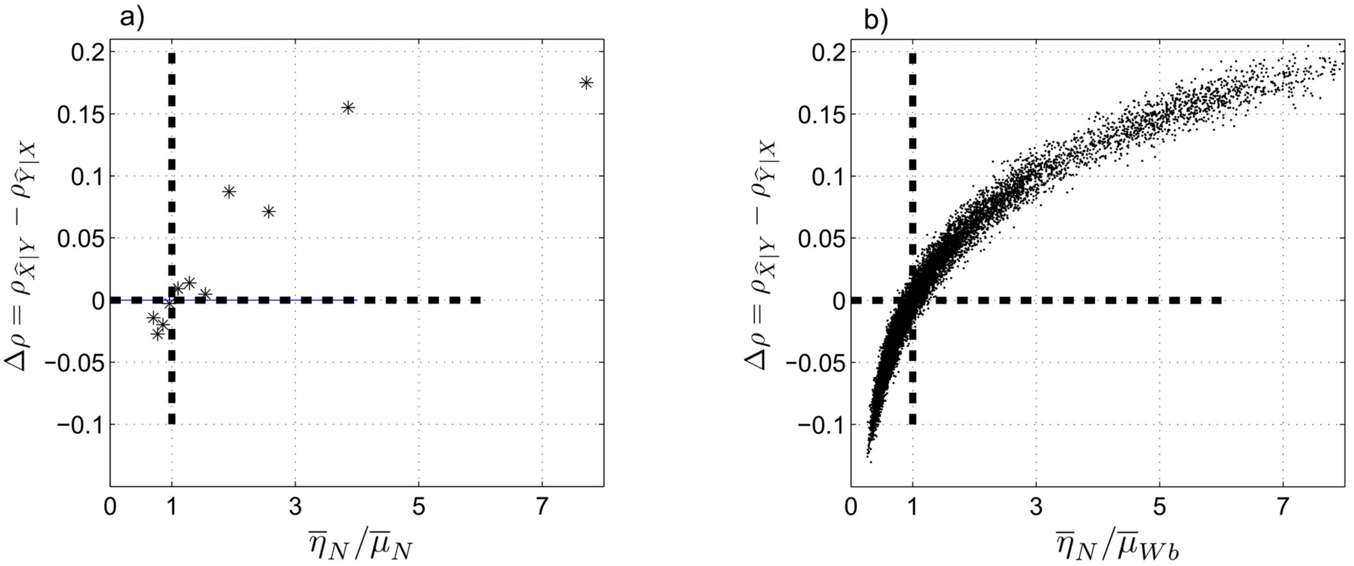

Fig 6. Case III: mixed normal–nonnormal coupling coefficients. (a) $\Delta\rho = \rho_{\hat{X}|Y} - \rho_{\hat{Y}|X}$ at L = 500 as a function of $\overline{\eta}_N/\overline{\mu}_{Wb}$. (b) 1000 independent realizations of Eq (12) and the corresponding $\Delta\rho$ at L = 500 as a function of $\overline{\eta}_N/\overline{\mu}_{Wb}$. $\overline{\eta}_N$ and $\overline{\mu}_{Wb}$ are the mean values of η_N and μ_{Wb} over the span of the time–series. $\Delta\rho$ = 0 and $\overline{\eta}_N/\overline{\mu}_N = 1$ are shown by dashed, bold lines.

doi:10.1371/journal.pone.0131226.g006

coefficients, except when the two sub–systems have almost equal coupling coefficients. We also note that in both cases II and III, detection of the leading sub–system is more reliable for larger values of $(\overline{\eta}/\overline{\mu})$.

In contrast to case II, we observe a nonlinear relationship between $\Delta\rho$ and $\overline{\eta}_N/\overline{\mu}_{Wb}$, i.e., the rate of change of $\Delta\rho$ decreases rapidly as $\overline{\eta}_N/\overline{\mu}_{Wb}$ increases. Thus, we see a faster saturation in Fig 6 compared to Fig 4. Also, non–symmetric distribution of the data points around the vertical line $\overline{\eta}_N/\overline{\mu}_{Wb} = 1$ in Fig 6b is due to the range of $\overline{\eta}_N/\overline{\mu}_{Wb}$ according to Eq (12), which has a longer tail on the right hand side ($\overline{\eta}_N/\overline{\mu}_{Wb} > 1$). If we plot $-\Delta\rho = \rho_{\hat{Y}|X} - \rho_{\hat{X}|Y}$ vs. $\overline{\mu}_{Wb}/\overline{\eta}_N$, we would see a similar trend as in Fig 6b, although some ranges are non–overlapping due to the asymmetric distribution of the coupling coefficients.

Similar to case II, we repeat the non–parametric one–sample Kolmogorov–Smirnov test and t-test for $\Delta\rho$ values of Fig 6a. In this case, except one data point that lies closest to the vertical axis $\overline{\eta}_N/\overline{\mu}_{Wb} = 1$, all other data points have t–values larger than the required minimum corresponding to a critical p–value of 0.01. Therefore, we can reject the null hypothesis and conclude that $\Delta\rho$ is significant at 0.01 level.

3.4 Case IV: Coupled Lorenz systems, temporally shifted and noisy signals

In this section, we investigate the ability of the CCM method for detecting the leading system in the presence of chronological uncertainties (temporal uncertainty between the two time–series) as well as additive Gaussian white noise. We simulate the chronological uncertainties by relative shifting of the two time–series. We take the signals from cases II and III and apply relative shifts of 2.5 and 5% of the full length of the time–series.

For the additive Gaussian white noise, we consider a normal random variable with zero mean and standard deviation set at 5 and 10% level of the standard deviation of the original

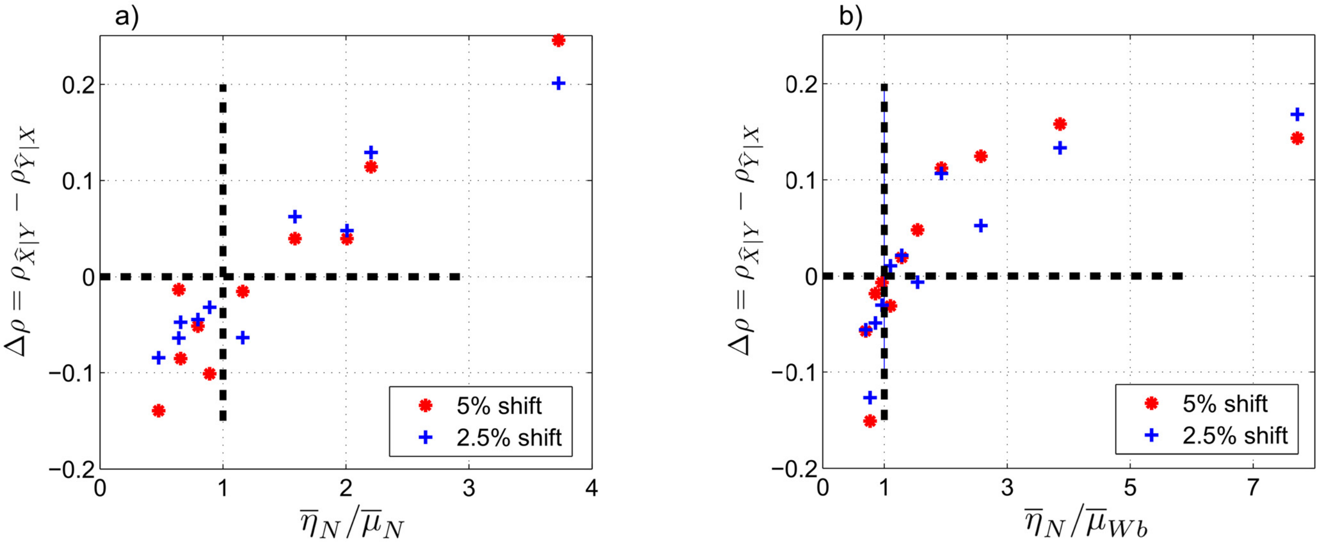

Fig 7. Case IV: experiments on signals of cases II and III with imposed temporal uncertainties. We consider temporal shifts corresponding to 2.5% and 5% of the full length of the time series in cases II and III. (a) $\Delta\rho$ at L = 500 as a function of $\bar{\eta}_N/\bar{\mu}_N$ for shifted signals of case II. (b) $\Delta\rho$ at L = 500 as a function of $\bar{\eta}_N/\bar{\mu}_{Wb}$ for shifted signals of case III. $\Delta\rho$ = 0 and $\bar{\eta}_N/\bar{\mu}_N = 1$ are shown by dashed, bold lines.

doi:10.1371/journal.pone.0131226.g007

signals in cases II and III. By this choice, the signal–to–noise ratios (SNR) are equal to 400 and 100 respectively, showing moderate noise levels.

As before, we compute $\Delta\rho$ with respect to the ratio of the averaged coupling coefficients, $(\bar{\eta}/\bar{\mu})$, under different conditions. Results of the experiments with chronological uncertainties and additive noise (see Figs 7 and 8) show the same features as in Figs 4a and 6a. For example, $\Delta\rho$ is negative when $(\bar{\eta}/\bar{\mu}) < 1$ and positive when $(\bar{\eta}/\bar{\mu}) > 1$, with few exceptional data points

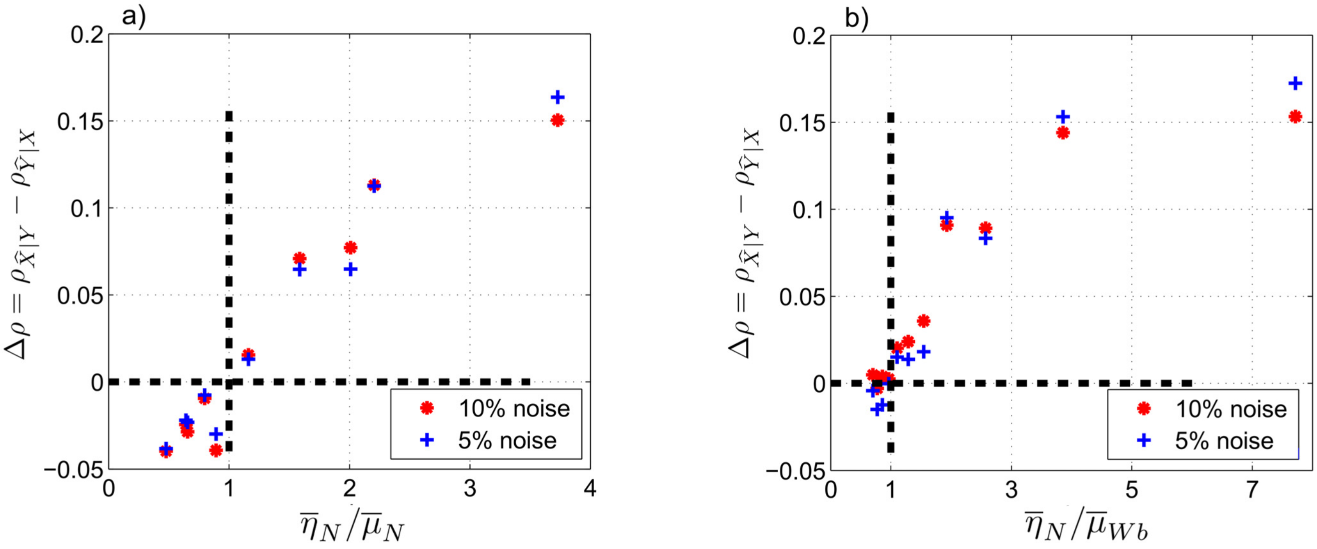

Fig 8. Case IV: experiments on signals of cases II and III with imposed additive Gaussian noise. We consider noise levels at 5% and 10% of the standard deviation of the original signals in cases II and III. (a) $\Delta\rho$ at L = 500 as a function of $\bar{\eta}_N/\bar{\mu}_N$ for signals of case II. (b) $\Delta\rho$ at L = 500 as a function of $\bar{\eta}_N/\bar{\mu}_{Wb}$ for signals of case III. $\Delta\rho$ = 0 and $\bar{\eta}_N/\bar{\mu}_N = 1$ are shown by dashed, bold lines.

doi:10.1371/journal.pone.0131226.g008

that are close to $(\bar{\eta}/\bar{\mu}) = 1$ in each figure. Also, we observe almost linear distribution of the data points in Figs 7a and 8a similar to the results of case II, Fig 4a, and nonlinear distribution of the data points in Figs 7b and 8b similar to case III, Fig 6a. These similarities show that the CCM results are robust to the imposed temporal uncertainties and additive white noise, especially for larger ratios of the coupling coefficients.

4 Summary

There is often interest in determining causal relationship between variables of a physical system. Establishing a method for causality analysis is hence of paramount importance [22]. Linear lead–lag analysis is commonly applied to underpin causal relationship when there are some evidences about the internal mechanisms of the system. But quite frequently, two signals show inconsistent lead–lag behavior in different time windows due to nonlinearities of the system. Therefore, lead–lag analysis cannot conclusively determine causality over the full time span of observed signals. Introducing Granger causality [10] was a turning point in the field of causality analysis. Based on Wiener's definition of causality, Granger proposed a practical method that is founded on the idea of predicting a variable both with and without a candidate driver. If forecast is significantly improved when the information of the candidate driver is included in the set of predictors, the Granger method concludes that the candidate signal is a driver. Transfer entropy was introduced as a measure of directional communication between elements of a system [3]. Later, it was shown that Granger causality and transfer entropy are equivalent for Gaussian variables [12]. Although information transfer measures provide important understanding about the directional interconnections in a system, they do not identify efficient causal relationships. It is shown that interventional methods and information flow can identify micro–level causal relationships [21]. Sugihara et al. [23] proposed a new idea for causality analysis, applicable to deterministic nonlinear systems, based on cross convergent mapping between shadowing manifolds. They successfully applied the CCM method to analyze causality in weakly coupled systems with constant coupling coefficients.

This study was inspired by identifying the leading element in systems that are speculated to have time–varying internal connections with probable change of dominant sub–system in different periods, for example, the earth system with its many interconnected sub–systems. For the first time, we addressed applicability of the CCM method to coupled systems with time–varying coupling coefficients and switching between dominant elements in different periods. We conducted numerical experiments with I) periodic–constant, II) normal, and III) mixed normal and non–normal coupling coefficients. In experiment IV, we imposed temporal uncertainties and additive noise to the observed time–series of cases II and III. We investigated whether the CCM method can identify the leading sub–system that has a larger average coupling coefficient over the entire span of the time–series.

Our main conclusions are:

1. If the averaged coupling coefficients are not approximately equal, i.e., $\bar{\eta} \not\approx \bar{\mu}$, and a leading system exists, then the CCM coefficient of the leading system is significantly larger than the CCM coefficient of the system with a smaller average coupling coefficient.
2. If the ratio of the average coupling coefficients is close to one $(\bar{\eta}/\bar{\mu} \approx 1)$, the leading system is not well–defined and the CCM method is not applicable.
3. The CCM results are quite robust to temporal uncertainties and moderate levels of additive Gaussian noise.

4. For normally distributed coupling coefficients (case II), a close–to–linear relationship between $\Delta\rho$ and the ratio of the average coupling coefficients is observed (in the range of the selected coefficients).

5. For mixed normally and non–normally distributed coupling coefficients (case III), a [saturating] nonlinear relationship between $\Delta\rho$ and the ratio of the average coupling coefficients is observed (in the range of the selected coefficients).

According to these observations, we conclude that when the ratio of the average coupling coefficients is not close to one, the CCM method can detect the leading sub–system in a set of two coupled systems with time–varying coupling coefficients—even in the presence of chronological uncertainties or additive Gaussian noise.

There are still questions regarding applicability of CCM to systems with time delays [lagged influences], different time scales, different embedding dimensions, non-identical sub–systems, nonlinear influences, and non–smooth manifolds. Application of CCM to high–dimensional systems and non–stationary signals demands future studies too. Sufficiency of the number of observed data points for a reliable CCM analysis is another question that remains open. All of these open questions call for potential extensions of our study.

We anticipate the CCM method can be employed to study causal relationships between variables of systems such as those in atmospheric science, biology, ecology, epidemiology, and sociology.

Acknowledgments

We greatly benefited from many discussions with Professors James Yorke, Brian Hunt and Shane Ross. We thank the reviewers for their insightful comments and suggestions.

Author Contributions

Conceived and designed the experiments: AEBM SM SGP EK. Performed the experiments: AEBM SM SGP EK. Analyzed the data: AEBM SM SGP EK. Contributed reagents/materials/analysis tools: AEBM SM SGP EK. Wrote the paper: AEBM SM SGP EK.

References

1. Chatfield C. The analysis of time series: an introduction. CRC press; 2013.
2. Nolte G, Ziehe A, Nikulin V, Schlögl A, Krämer N, Brismar T, et al. Robustly Estimating the Flow Direction of Information in Complex Physical Systems. Physical Review Letters. 2008 June; 100:234101. Available from: http://doc.ml.tu-berlin.de/causality. doi: 10.1103/PhysRevLett.100.234101 PMID: 18643502
3. Schreiber T. Measuring Information Transfer. Phys Rev Lett. 2000 Jul; 85:461–464. doi: 10.1103/PhysRevLett.85.461 PMID: 10991308
4. Vicente R, Wibral M, Lindner M, Pipa G. Transfer entropy—a model-free measure of effective connectivity for the neurosciences. Journal of Computational Neuroscience 2011; 30(1):45–67. doi: 10.1007/s10827-010-0262-3 PMID: 20706781
5. Ding M, Chen Y, Bressler SL. 17 Granger causality: basic theory and application to neuroscience. Handbook of time series analysis: recent theoretical developments and applications. 2006;p. 437.
6. Bauer M, Cox JW, Caveness MH, Downs JJ, Thornhill NF. Finding the Direction of Disturbance Propagation in a Chemical Process Using Transfer Entropy. Control Systems Technology, IEEE Transactions on. 2007 Jan; 15(1):12–21. Available from: http://ieeexplore.ieee.org/stamp/stamp.jsp?tp=&arnumber=4039335. doi: 10.1109/TCST.2006.883234
7. Lizier JT, Prokopenko M, Zomaya AY. Local information transfer as a spatiotemporal filter for complex systems. Phys Rev E. 2008 Feb; 77:026110. doi: 10.1103/PhysRevE.77.026110

8. Wibral M, Pampu N, Priesemann V, Siebenhühner F, Seiwert H, Lindner M, et al. Measuring Information-Transfer Delays. PLoS ONE. 2013 02; 8(2):e55809. doi: 10.1371/journal.pone.0055809 PMID: 23468850
9. Wiener N. The theory of prediction. Modern mathematics for engineers. 1956; 1:125–139.
10. Granger CWJ. Investigating Causal Relations by Econometric Models and Cross-spectral Methods. Econometrica. 1969; 37(3):pp. 424–438. doi: 10.2307/1912791
11. Granger CWJ. Testing for causality: A personal viewpoint. Journal of Economic Dynamics and Control. 1980; 2(0):329–352. doi: 10.1016/0165-1889(80)90069-X
12. Barnett L, Barrett AB, Seth AK. Granger Causality and Transfer Entropy Are Equivalent for Gaussian Variables. Phys Rev Lett. 2009 Dec; 103:238701. doi: 10.1103/PhysRevLett.103.238701 PMID: 20366183
13. Hlaváčková-Schindler K, Paluš M, Vejmelka M, Bhattacharya J. Causality detection based on information-theoretic approaches in time series analysis. Physics Reports. 2007; 441(1):1–46. Available from: http://www.sciencedirect.com/science/article/pii/S0370157307000403. doi: 10.1016/j.physrep.2006.12.004
14. Bressler SL, Seth AK. Wiener-Granger Causality: A well established methodology. NeuroImage. 2011; 58(2):323–329. Available from: http://www.sciencedirect.com/science/article/pii/S1053811910002272. doi: 10.1016/j.neuroimage.2010.02.059 PMID: 20202481
15. Barnett L, Seth AK. The MVGC multivariate Granger causality toolbox: A new approach to Granger-causal inference. Journal of Neuroscience Methods. 2014; 223(0):50–68. Available from: http://www.sciencedirect.com/science/article/pii/S0165027013003701. doi: 10.1016/j.jneumeth.2013.10.018 PMID: 24200508
16. Chen Y, Rangarajan G, Feng J, Ding M. Analyzing multiple nonlinear time series with extended Granger causality. Physics Letters A. 2004; 324(1):26–35. Available from: http://www.sciencedirect.com/science/article/pii/S0375960104002403. doi: 10.1016/j.physleta.2004.02.032
17. Marinazzo D, Pellicoro M, Stramaglia S. Kernel Method for Nonlinear Granger Causality. Phys Rev Lett. 2008 Apr; 100:144103. doi: 10.1103/PhysRevLett.100.144103 PMID: 18518037
18. Hiemstra C, Jones JD. Testing for Linear and Nonlinear Granger Causality in the Stock Price-Volume Relation. The Journal of Finance. 1994; 49(5):1639–1664. Available from: http://dx.doi.org/10.1111/j.1540-6261.1994.tb04776.x. doi: 10.2307/2329266
19. Cover TM, Thomas JA. Elements of information theory. John Wiley & Sons; 2012.
20. Ay N, Polani D. Information flows in causal networks. Advances in Complex Systems. 2008; 11(01):17–41. doi: 10.1142/S0219525908001465
21. Lizier JT, Prokopenko M. Differentiating information transfer and causal effect. The European Physical Journal B. 2010; 73(4):605–615. doi: 10.1140/epjb/e2010-00034-5
22. Pearl J. Causality: models, reasoning and inference. vol. 29. Cambridge Univ Press; 2000.
23. Sugihara G, May R, Ye H, Hsieh Ch, Deyle E, Fogarty M, et al. Detecting Causality in Complex Ecosystems. Science. 2012; 338(6106):496–500. Available from: http://www.sciencemag.org/content/338/6106/496.abstract. doi: 10.1126/science.1227079 PMID: 22997134
24. Deyle ER, Fogarty M, Hsieh Ch, Kaufman L, MacCall AD, Munch SB, et al. Predicting climate effects on Pacific sardine. Proceedings of the National Academy of Sciences. 2013; 110(16):6430–6435. Available from: http://www.pnas.org/content/110/16/6430.abstract. doi: 10.1073/pnas.1215506110
25. van Nes, EH, Scheffer, M, Brovkin, V, Lenton, TM, Ye, H, Deyle, E, et al. Causal feedbacks in climate change. Nature Climate Change. 2015;p. 445–448. Available from: http://www.nature.com/nclimate/journal/v5/n5/abs/nclimate2568.html#supplementary-information.
26. Monnin E, Indermühle A, Dällenbach A, Flückiger J, Stauffer B, Stocker TF, et al. Atmospheric CO2 Concentrations over the Last Glacial Termination. Science. 2001; 291(5501):112–114. Available from: http://www.sciencemag.org/content/291/5501/112.abstract. doi: 10.1126/science.291.5501.112 PMID: 11141559
27. Caillon N, Severinghaus JP, Jouzel J, Barnola JM, Kang J, Lipenkov VY. Timing of Atmospheric CO2 and Antarctic Temperature Changes Across Termination III. Science. 2003; 299(5613):1728–1731. Available from: http://www.sciencemag.org/content/299/5613/1728.abstract. doi: 10.1126/science.1078758 PMID: 12637743
28. Shakun JD, Clark PU, He F, Marcott SA, Mix AC, Liu Z, et al. Global warming preceded by increasing carbon dioxide concentrations during the last deglaciation. Nature. 2012; 484(7392):49–54. doi: 10.1038/nature10915 PMID: 22481357
29. Lorenz EN. Deterministic nonperiodic flow. Journal of the atmospheric sciences. 1963; 20(2):130–141. doi: 10.1175/1520-0469(1963)020%3C0130:DNF%3E2.0.CO;2

30. Yang SC, Baker D, Li H, Cordes K, Huff M, Nagpal G, et al. Data assimilation as synchronization of truth and model: Experiments with the three-variable lorenz system. Journal of the atmospheric sciences. 2006; 63(9):2340–2354. doi: 10.1175/JAS3739.1
31. Takens F. Detecting strange attractors in turbulence. In: Dynamical systems and turbulence, Warwick 1980. Springer; 1981. p. 366–381.
32. Sauer T, Yorke J, Casdagli M. Embedology. Journal of Statistical Physics. 1991; 65(3–4):579–616. doi: 10.1007/BF01053745
33. Cao L. Practical method for determining the minimum embedding dimension of a scalar time series. Physica D: Nonlinear Phenomena. 1997; 110(1–2):43–50. Available from: http://www.sciencedirect.com/science/article/pii/S0167278997001188. doi: 10.1016/S0167-2789(97)00118-8
34. Deyle ER, Sugihara G. Generalized Theorems for Nonlinear State Space Reconstruction. PLoS ONE. 2011 03; 6(3):e18295. doi: 10.1371/journal.pone.0018295 PMID: 21483839
35. Fraser AM, Swinney HL. Independent coordinates for strange attractors from mutual information. Phys Rev A. 1986 Feb; 33:1134–1140. doi: 10.1103/PhysRevA.33.1134 PMID: 9896728
36. Abarbanel H. Analysis of observed chaotic data. Springer; 1996.
37. Farmer JD, Sidorowich JJ. Predicting chaotic time series. Phys Rev Lett. 1987 Aug; 59:845–848. doi: 10.1103/PhysRevLett.59.845 PMID: 10035887
38. Hunt BR, Ott E, Yorke JA. Differentiable generalized synchronization of chaos. Phys Rev E. 1997 Apr; 55:4029–4034. doi: 10.1103/PhysRevE.55.4029
39. Pecora LM, Carroll TL, Johnson GA, Mar DJ, Heagy JF. Fundamentals of synchronization in chaotic systems, concepts, and applications. Chaos. 1997; 7(4):520–543. doi: 10.1063/1.166278 PMID: 12779679
40. Peña M, Kalnay E. Separating fast and slow modes in coupled chaotic systems. Nonlinear Processes in Geophysics. 2004 Jul; 11(3):319–327. Available from: https://hal.archives-ouvertes.fr/hal-00302339. doi: 10.5194/npg-11-319-2004
41. Rinne H. The Weibull distribution: a handbook. CRC Press; 2008.
